# Endothelial perturbations and therapeutic strategies in normal tissue radiation damage

**DOI:** 10.1186/s13014-014-0266-7

**Published:** 2014-12-18

**Authors:** Elina Korpela, Stanley K Liu

**Affiliations:** Biological Sciences, Sunnybrook Research Institute and Odette Cancer Centre, Sunnybrook Health Sciences Centre, 2075 Bayview Ave., Toronto, M4N 3M5 Canada; Department of Medical Biophysics, University of Toronto, 101 College St., Toronto, M5G 1L7 Canada; Department of Radiation Oncology, University of Toronto, 149 College St., Toronto, M5T 1P5 Canada

**Keywords:** Radiotherapy, Acute radiation toxicity, Endothelial cell, Microvasculature, Radioprotection, Cell death, Inflammation, Senescence

## Abstract

Most cancer patients are treated with radiotherapy, but the treatment can also damage the surrounding normal tissue. Radiotherapy side-effects diminish patients’ quality of life, yet effective biological interventions for normal tissue damage are lacking. Protecting microvascular endothelial cells from the effects of irradiation is emerging as a targeted damage-reduction strategy. We illustrate the concept of the microvasculature as a mediator of overall normal tissue radiation toxicity through cell death, vascular inflammation (hemodynamic and molecular changes) and a change in functional capacity. Endothelial cell targeted therapies that protect against such endothelial cell perturbations and the development of acute normal tissue damage are mostly under preclinical development. Since acute radiation toxicity is a common clinical problem in cutaneous, gastrointestinal and mucosal tissues, we also focus on damage in these tissues.

## Introduction

Despite technology-driven improvements in cancer radiotherapy (RT), normal tissue radiation toxicities remain a significant clinical concern [1]. They can influence treatment outcomes, patient quality of life and survivorship. For example, early skin toxicities which develop within the first few weeks of RT commencement tend to be transient. Nonetheless, approximately 30% of breast cancer patients and 60% of head and neck cancer patients treated with RT develop painful, infection-prone severe epithelial barrier breakdown (desquamation) [2,3]. This can complicate tissue reconstruction efforts [4] or necessitate treatment interruptions, which have been found to compromise tumour control or cure [5]. Furthermore, late radiation toxicity occurs months to years following RT, and can result in permanently debilitating organ dysfunction such as cardiovascular diseases (CVDs) [6].

The three categories of radiation protectors include radioprotectants, radiomitigators and therapeutics. These are administered either before radiation exposure, after radiation exposure but before damage manifestation, or after damage manifestation, respectively. In the clinic, acute toxicities such as desquamation are managed non-specifically with mitigative or therapeutic strategies. Medicated ointments and dressings are in use with conflicting or minimal evidence and they do not prevent the manifestation of the problematic damage that impedes patient wellbeing [7]. On the other hand, directly minimizing the biological determinants of the damage is an approach to preventing these impediments. Amifostine is the only targeted radioprotectant with enough clinical evidence to support its use. However, it has practical limitations and a significant toxicity profile [8]. Several biological mechanisms of normal tissue radiation protection are well explored at the preclinical level, but the endothelial cell (EC) compartment is now also emerging as an attractive target for radiation protection.

We argue that protecting microvascular endothelial cells from radiation-induced perturbations, or disruptions to the normal homeostatic or angiogenic state, ultimately protects the normal tissue from radiation damage. These perturbations include EC death, vascular inflammation (hemodynamic and molecular changes) and loss of functional capacity.

## Review

### General mechanisms of radiation protection

General mechanisms of radiation protection include the use of antioxidants, modulation of cell death, inflammation suppression and promotion of wound healing. These have recently been thoroughly reviewed [9]. Nevertheless, notable examples of targeted agents under preclinical and clinical investigation are discussed while outlining the general process of ionizing radiation (IR)-induced damage.

A direct radioprotectant reduces the amount of cellular DNA damage so that a cell can remain healthy and functional. The most well-studied radioprotectant, amifostine, contains a free radical-scavenging sulfhydryl group [10], competes with oxygen to reduce permanent DNA damage fixation and increases expression of an endogenous detoxifying enzyme manganese superoxide dismutase [11], thereby reducing double stranded break accumulation [12,13] and genomic instability [14]. Initially, amifostine was particularly promising for improving the therapeutic ratio of cancer RT due to its preferential accumulation in normal tissue rather than cancerous tissue. Even still, concerns with toxic side-effects and potential cancer recurrence discourage the implementation of this class of radioprotectants during cancer RT [15].

If the IR-induced DNA damage cannot be repaired, the cell will proceed with clonogenic or reproductive death that can include programmed cell death (apoptosis), mitotic catastrophe or senescence, leading to cellular hypoplasia and the observed symptoms of clinical radiotoxicity. Pifithrin-α, an inhibitor of the p53-mediated apoptotic pathway that is activated by DNA damage and genotoxic stress, reduces mortality of mice after total body irradiation (TBI) [16]. There is concern that preventing normal cells that harbor relevant DNA damage from dying will increase the likelihood of their malignant transformation.

After radiation exposure, the master regulator of inflammation known as nuclear factor kappa-light-chain-enhancer of activated B cells (the NF-ҡB complex) is released and translocates to the nucleus to initiate the transcriptional program of pro-survival factors and cytokines. The mounting inflammatory damage may provoke unnecessary tissue injury severity above and beyond the initial insult consisting of DNA damage and subsequent hypoplasia. Although the activity of NF-ҡB is generally described as detrimental, surprisingly, there is support to the contrary. For example, the bacterial flagellin protein derivative CBLB502 that activates NF-ҡB signaling (by activating Toll-like receptor 5) can suppress IR-induced apoptosis in the gastrointestinal (GI) tract [17]. More recently, CBLB502 also prevented murine mucositis [18]. Strategies that permit inflammation but modulate a certain aspect of the process may be a more promising avenue for this class of radiation protectants.

With enough IR-induced epithelial progenitor cell apoptosis, tissue barrier function fails resulting in desquamation/ulceration and exacerbation of inflammation. Tissue repopulation by the stimulation of progenitor cell proliferation or bone marrow (BM) cell recruitment are approaches to reduce barrier function disruption and to promote wound healing. Palifermin, a human recombinant keratinocyte growth factor that stimulates salivary gland stem cell proliferation, reduced the number of treatment interruptions overall and the duration and incidence of mucositis among hyperfractionated patients [19]. Granulocyte colony stimulating factor (G-CSF, which stimulates neutrophil-trafficking from the BM) is already commercially used to treat neutropenia during chemotherapy. G-CSF treatment reduced skin radiation damage severity in mice [20] and hastened moist desquamation healing in cancer patients treated with RT [21]. Additionally, the profibrogenic cytokine transforming growth factor-β (TGF-β) [22] is generally considered to be detrimental to radiation wound healing speed, subsequent tissue remodeling and late toxicity risk. Indeed, knockdown of its downstream mediator Smad3 accelerated healing of radiation wounds [23]. Additionally, haploinsufficiency of the TGF-β co-receptor endoglin was protective against late kidney fibrosis after radiation challenge [24] but not for myocardial fitness [25].

### Microvasculature as a mediator of radiation damage

The vasculature consists of large, medium and small-diameter vessels, the latter of which is the microvasculature. The microvascular arterioles, capillaries and venules all serve to deliver nutrients and oxygen and remove metabolic wastes from the parenchymal cells they supply. These are lined by a single inner layer of ECs supported by pericytes or smooth muscle cells. EC dysfunction is already believed to be a critical contributor of late radiation tissue damage in certain tissues. For example, radiation damage to the skin or mucosal vasculature can lead to telangiectasia, which is a pruned vascular network of fragile, enlarged vessels prone to bleeding and with limited functional capacity (reviewed in reference [26]). Radiation damage to large vessels also predisposes patients to CVDs, a subject which was recently reviewed (see reference [27]), but also briefly addressed within this work. Yet there is increasing evidence that microvascular events also contribute to acute damage development. In fact, more recent studies suggest that protecting ECs against such perturbations using genetic or pharmacologic strategies protects GI, mucosal, cardiac and skin tissues from radiation damage. Unfortunately, many informative EC radiation protection studies have utilized TBI, which limits their clinical relevance.

#### EC loss following irradiation

##### Mechanisms of EC loss

*In vivo*, capillaries begin to dissipate as early as 1 day after low or high-dose irradiation, may rupture and are lost more readily than the larger arterioles and venules [28,29]. A wave of microvascular EC apoptosis has been reported to begin 1 to 24 hours following IR exposure *in vitro* [30] and *in vivo* in the lung [31], central nervous system [32], GI tract [33], parotid glands [34] and myocardium [35]. Starting around 30 days, small vessel density decreases [26].

It is known that ECs are vulnerable to acid sphingomyelinase (ASMase)-mediated cell death [36]. Stress can induce an early pro-apoptotic signal through ASMase-mediated ceramide platform formation on their outer membranes [37]. This signal may influence tissue progenitor cell death, as demonstrated by Lu and colleagues [38]. They found that normal neural progenitor cells transplanted into ASMase-deficient mice did not undergo their expected apoptosis when irradiated in their new environment. Irradiated ECs are also susceptible to mitochondrial Bak/Bax-mediated cell death, which are activated by p53 activity [39].

##### Protecting ECs from apoptosis protects normal irradiated tissue

Strategies to protect ECs from apoptosis have been associated with improvements in preclinical radiation toxicity model outcomes. For example, early radiation-induced apoptosis of ECs is inhibited by basic fibroblast growth factor (bFGF) *in vitro* [40] and *in vivo* [41]. The effect has been associated with improved survival of mice with radiation pneumonitis [31]. In a landmark study of radiation-induced GI toxicity, Paris *et al.* demonstrated that bFGF treatment and ASMase deficiency both decreased EC apoptosis and improved survival from GI syndrome following TBI [33]. This effect was also observed with an EC-protecting Angiopoietin-1-based construct, which promotes EC viability and stability [42]. Schuller and colleagues challenged the view that the early EC apoptosis initiates radiation-induced GI syndrome with the finding that selective *in vivo* irradiation of ECs did not evoke an apoptotic response in ECs that could initiate GI failure, but did in epithelial cells [43]. More recently, a ceramide platform formation-blocking antibody and EC plasminogen activator I-1 deficiency (a p53 target gene product that normally prevents insoluble fibrin breakdown) both prevented EC death and delayed lethality due to radioenterogastritis in mice [44,45], again supporting the idea that EC death is a critical aspect of radiation injury. Lastly, although dominated by GI models of radiotoxicity, this radioprotection strategy extends to other systems. Mice lacking Bak and Bax only in ECs experienced faster hematopoietic stem cell recovery after 3 Gy TBI [46]. Additionally, mice treated with an EC-protecting anti-CD47 (a thrombospondin-1 receptor that normally promotes vascular perturbations) morpholino experienced less skin damage from a 25 Gy dose of IR [47].

Although genetic knockout of p53 downstream components decreases EC apoptosis and protects the tissue, direct p53 knockouts have yielded surprising findings. After 12 Gy irradiation of the heart, mice that lacked p53 in ECs displayed increased EC death and decreased microvascular density 4 weeks post IR, succumbing to heart failure around 8 weeks unlike their p53-wild-type counterparts [35]. Additionally, these mice die off rapidly a month after TBI from late GI syndrome [48]. This suggests that an EC p53 deficit is detrimental to irradiated EC survival and exacerbates late reactions. However, the difference in radioprotective effects between p53 and downstream target knockouts remains to be explained.

#### Vascular inflammatory responses induced by irradiation

##### Hemodynamic changes

The inflammatory response of the vasculature to IR has been described over the past 50 years largely by focusing on parameters such as vessel diameter and blood flow. Within hours after radiation exposure, the vasculature becomes leaky [49], although the degree to which ECs of various vessel types become permeable varies *in vitro* [50]. Investigation of early time points following rodent skin radiation exposure have yielded a bi-phasic response in vascular perfusion parameters as measured by hyperspectral imaging [51] and isotopic labeling of red blood cells and microspheres [52]. This mirrors the early transient erythema and a secondary delayed erythema observed in the clinic [53,54]. The opposite has also been reported: blood flow measured by laser Doppler in irradiated hamster parotid gland and mouse skin decreased from baseline up to 2 or 3 weeks [34,55]. Irradiation of hamster cheek pouch muscle also caused microvascular red blood cell velocity to decrease as determined by intravital microscopy [56]. These incongruent findings may arise from the different tissues, IR doses and imaging time points under study. Furthermore, different imaging techniques are amenable to assessing diverse subsets of blood vessels owing to subjective definitions or disparate technique sensitivities. Although these findings highlight the notion that the vasculature changes functionally in response to IR, measuring perfusion or blood flow alone may not be a robust measure of IR-induced hemodynamic changes. In fact, a thorough investigation of numerous parameters may be required to understand the overall changing microvascular landscape, as argued by Archambeau *et al.* [26]. Observing functional vascular hemodynamic changes using imaging modalities following irradiation could be useful to detect differences between control and EC-protecting agents.

Unlike the variability in reports on the effect of radiation on perfusion and blood flow, reports monitoring oxygenated hemoglobin (oxyHb) through diffuse reflectance spectroscopy, although fewer, are in better agreement. OxyHb is a measure of inflammation and correlates with topical irritant dose [57] and erythema severity [58]. Chin *et al.* [51] and others [59,60] have found that oxyHb increases in irradiated rodent skin. Similar measurements have been reported as useful indicators of radiodermatitis in clinical studies [61,62]. We reported that Vasculotide reduced the severity of IR-induced radiodermatitis in mice, and this was accompanied by lower oxyHb measurements than in irradiated controls [63]. The agreement in the literature on the irradiation-induced rise in oxyHb may result from quantification from all vessel sizes in the area (rather than a subset). Additionally, it indicates inflammation, which is a pronounced effect of IR, and might not be influenced as strongly by variable degrees of vessel loss. Therefore, oxyHb quantification may be a reliable method of measuring inflammation as a measure of hemodynamic changes.

##### EC inflammatory activation

Although inflammation is orchestrated by several cell types, the vascular EC lining is a key modulator of the response (reviewed in reference [64]). Classical inflammation begins with the secretion of tumour necrosis factor α (TNF-α) and IL-1 by damaged cells or immune cells. These cytokines cause NF-ҡB to be released and initiate a pro-inflammatory transcriptional program in ECs, which then secrete chemoattractants and express adhesion molecules. Neutrophils bind the adhesion molecules and undergo transendothelial diapedesis into injured tissue, where they can undergo respiratory burst and contribute to the progression of the inflammatory response in complex ways, as also recently reviewed [65]. Numerous studies have been conducted detailing EC cytokine production and cytokine effects on EC responses. Recently, Halle and colleagues showed for the first time that NF-ҡB is up-regulated in irradiated arteries of patients treated with RT months or years before [66]. The prolongation of inflammation may create a milieu conducive to the development of chronic IR-induced pathologies such as fibrosis and atherosclerosis.

##### Dampening EC inflammatory activation protects normal irradiated tissue

Reduced pro-inflammatory cytokine, chemokine, and EC adhesion molecule levels are usually associated with better radiotoxicity outcomes. Genetic knockout studies have revealed the importance of a few key players in radiation damage presentation.

i) Cytokine players

IL-1 is produced immediately following tissue irradiation [67]. It is made in two forms, IL-1α and β, mainly by keratinocytes in irradiated skin, but also by ECs (among other cells). They stimulate EC adhesion molecule presentation. Combinational IL-1α and IL-1β knockout mice demonstrated subdued radiodermatitis compared to either single cytokine knockout alone [68].

IL-6 is a pleiotropic cytokine with both pro- and anti-inflammatory effects [69]. It is an important acute inflammatory phase mediator (e.g. causing fever) but uncontrolled overproduction may also contribute to inflammatory diseases [70]. ECs (among other cells) secrete IL-6 through NF-ҡB activation following irradiation [71] and following IL-1 and TNF-α stimulation. One of its direct effects on ECs is the induction of intercellular adhesion molecule 1 (ICAM-1) expression [72]. IL-6 may also enhance hematopoietic cell recovery following TBI [73] especially in combination with G-CSF [74]. The potent anti-inflammatory effects of pravastatin were also observed on ECs *in vitro* through reduced IL-6 and IL-8/CXCL8 levels [75].

Human IL-8/CXCL8 is a chemoattractant for neutrophil chemotaxis. Its murine homologues are KC/CXCL1, MIP-2/CXCL2 and LIX/CXCL5, which vary temporally, spatially and in intensity when released in response to insults [76]. Human umbilical vein ECs (HUVECs) irradiated *in vitro* secrete IL-8/CXCL8 [77]. As expected, antagonizing MIP-2/CXCL2 cognate receptors CXCR1/CXCR2 improved survival in a mouse model of radiation-induced alveolitis [78].

TNF-α is a potent pro-inflammatory cytokine that induces EC permeability [79]. It is expressed immediately and cyclically after IR. TNF-α knockout mice experienced less severe radiation pneumonitis than wild-type mice [80].

Among the most commonly studied targets in radiopathology is TGF-β due to its involvement in chronic inflammation, fibrosis and late toxicities. Indeed, a small molecule TGF-β inhibitor mitigated 20 Gy-induced mouse lung fibrosis [81] and knockout of its canonical downstream mediator Smad3 attenuated capsular contracture of a prosthetic breast implant mouse model [82]. Secreted by various cells including fibroblasts and ECs, TGF-β is also a chemoattractant for a list of immune cells, and Smad3 knockdown reduced IR-induced skin inflammation [83]. Antagonizing the canonical TGF-β signaling pathway with Smad7 overexpression conferred striking early radiation protection of mouse oral mucosa [84].

ii) Adhesion molecule presentation

Many of these aforementioned cytokines induce EC expression of adhesion molecules such as ICAM-1, vascular cell adhesion molecule-1 (VCAM-1), E-selectin and P-selectin. Hallahan and Virudachalam demonstrated the importance of IR-induced microvascular EC ICAM-1 expression by showing that an ICAM-1 antibody treatment and ICAM-1 knockout rendered mice resistant to radiation pneumonitis [85]. Similarly, Holler *et al.* reported that pravastatin treatment mitigated 40 Gy acute skin radiation damage severity through diminished EC activation (ICAM-1 expression, etc.) and less neutrophil recruitment [86]. These results demonstrate that suppressing aspects of the inflammatory response to radiation, especially in regard to EC activation, can attenuate damage to normal tissues.

iii) Neutrophil presence

ECs are central to controlling inflammatory reactions at least partially through their gatekeeper function for neutrophil recruitment [65]. Activated ECs enable neutrophils to attach and undergo transendothelial migration into the tissue. The neutrophil respiratory burst releases myeloperoxidase, which produces ROS, and is normally important for pathogen clearance. The role of neutrophils in health and disease is growing in complexity [87], yet, neutrophil recruitment is generally considered to be detrimental in diseased tissue not under microbial attack. The association between decreased neutrophil counts (weeks after IR exposure) and better tissue outcomes is also the prevailing observation in skin radiation studies [23,68,83,84,86,88]. Mast cell presence, which is usually important in allergic reactions, is also lower in tissues with less severe radiation damage [89,90]. Interestingly, there are also reports associating extremely heightened but short-lived neutrophil recruitment with better tissue outcomes, but even these show reduced neutrophil counts at later time points [20,90].

#### Deterioration of EC function after irradiation

##### Induction of senescence

Various reports demonstrate that irradiation induces premature endothelial cell cycle arrest, or senescence [91,92]. The EC senescent phenotype consists of inflammatory activation, loss of proliferative capacity and other dysfunctional characteristics [93]. IR-induced senescence has also been associated with diminished pro-survival phosphatidylinositol-4,5-bisphosphate 3-kinase (PI3K) pathway signaling in HUVECs [92] and in ECs harvested from a range of human organs [94]. This IR-induced EC senescence and loss of function may contribute to the development of complications in populations treated with RT.

##### Preventing EC dysfunction protects normal irradiated tissue

Despite the requirement for slow EC turnover rates in normal tissue, irradiated ECs may fail to maintain an adequate EC population number by proliferation. For example, 25 Gy rat cranial irradiation lead to a gradual loss in EC density, followed by a proliferative burst after six months, but EC numbers fell drastically soon after giving way to necrosis [95].

Wounds in irradiated tissues take longer to heal than in non-irradiated tissues [96]. During the proliferative phase of acute wound healing, the damaged tissue is replaced by granulation tissue (rich in microvasculature and fibroblasts) and supplies all the cells involved in wound repair with oxygen and nutrients [97]. This suggests that irradiated vasculature may respond inadequately as it generates granulation tissue for wound healing. Evidence for this arises from *in vitro* experiments where irradiated HUVECs form fewer tubules in angiogenic assays [98,99] and have diminished proliferation and migration rates compared to non-irradiated controls [99]. Furthermore, the vascular beds of mouse skin exhibited reduced VEGF-induced angiogenesis after exposure to 25 Gy IR [99]. This dysfunction in angiogenesis could be rescued by treating with a small molecule inhibitor of the TGF-β receptor.

Patients treated with RT for breast cancer, Hodgkin’s lymphoma or childhood cancers are at increased risk to develop CVDs [100]. Aleman and colleagues observed a three to five-fold higher incidence of CVDs in patients treated previously for Hodgkin’s lymphoma and followed for a median of 18 years [6]. The IR-induced senescent state of ECs lining large vessels in irradiated fields may be one of the underlying conditions promoting CVD development. Indeed, the link between EC senescence and atherosclerosis has been established [101]. Moreover, preclinically, cardiac irradiation in the context of high cholesterol levels causes EC dysfunction and accelerates coronary atherosclerosis [102] which may lead to a myocardial infarct. Mouse models of radiation-induced cardiotoxicity show that although much of EC function is disrupted by IR, cardiac function is only modestly affected, suggesting a compensatory mechanism [25,102,103]. Lastly, irradiation (and senescence) reduces the endothelial response to vasodilating stimuli [104]. Interestingly, blood vessels in irradiated mouse hind limbs treated with an anti-CD47 morpholino retain this important function [47].

## Conclusions

**Figure 1:**
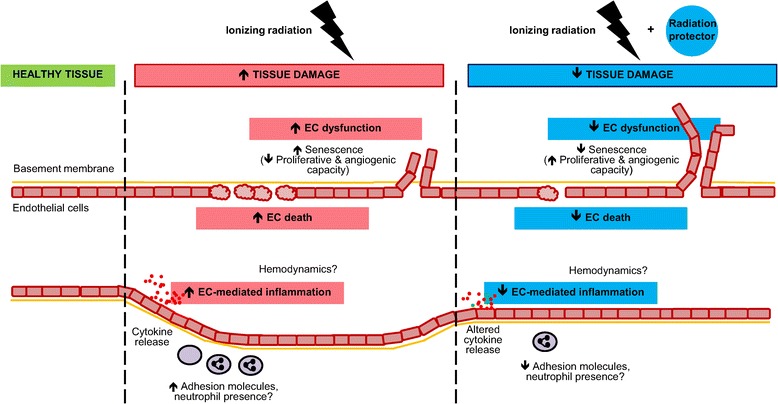
**Microvasculature as a mediator of (acute) IR damage and as a target for radiation protection.** Tissues that are exposed to a high enough dose of IR develop damage and undergo alterations. In the acute setting, IR induces EC loss through apoptosis and other mechanisms. It also affects vascular inflammatory responses in several ways. ECs become activated, expressing cell surface adhesion molecules and enabling neutrophil transendothelial migration. The timing of heightened neutrophil presence may be important for their effect on tissue damage. Additionally, cytokines are secreted and orchestrate further inflammatory responses. There is no clear consensus in the literature on the kind of hemodynamic changes that ensue, although it is known that vascular tone is lost over time. IR exposure also induces senescence which reduces EC proliferative and angiogenic capacity and causes a chronic pro-inflammatory phenotype. However, treating the tissue with a radioprotectant, radiomitigator or therapeutic that counters the microvascular-mediated damage development can result in reduced normal tissue damage.

The microvasculature is emerging as a biologically targetable compartment in the field of normal tissue radiation protection during RT. On the whole, preclinical radiation damage models support the notion that EC perturbations following irradiation contribute to the developing acute injury. The concept of the microvasculature as a mediator of acute radiation damage is illustrated in Figure 1. Conflicting findings may be attributed to the unique limitations of experimental methodologies or differing contexts of microvascular ECs in distinct organs. Indeed, it is known that ECs are diverse in their molecular responses and characteristics [105,106]. Additionally, it is conceivable that the tumor microenvironment could influence the response of normal tissue vasculature to irradiation [9,107], and thus future studies should account for this possibility.

Preclinically, some of these IR-induced perturbations can be countered; such therapeutics may translate into clinically beneficial treatments. EC-protecting agents may be most relevant for patients with pre-existing microvascular dysfunction (e.g. diabetes, obesity) and increased risk of radiotoxicity [108,109]. As all radiation protection strategies may not be suitable for all cancer RT regimens, the EC compartment may serve as an additional or alternative radiotherapeutic target to reduce the burden of acute normal tissue toxicity in cancer patients.

## Abbreviations

ASMase: Acid sphingomyelinase
bFGF: Fibroblast growth factor
BM: Bone marrow
CVD: Cardiovascular disease
EC: Endothelial cell
GI: Gastrointestinal
G-CSF: Granulocyte colony stimulating factor
HUVEC: Human umbilical vein EC
ICAM-1: Intercellular adhesion molecule 1
IR: Ionizing radiation
NF-ҡB: Nuclear factor kappa-light-chain-enhancer of activated B cells
oxyHb: Oxygenated haemoglobin
PI3K: Phosphatidylinositol-4,5-bisphosphate 3-kinase
RT: Radiation therapy
TBI: Total body irradiation
TGF-β: Transforming growth factor- β
TNF-α: Tumour necrosis factor α
VCAM-1: Vascular cell adhesion molecule 1
